# piR-823 tale as emerging cancer-hallmark molecular marker in different cancer types: a step-toward ncRNA-precision

**DOI:** 10.1007/s00210-024-03308-z

**Published:** 2024-08-05

**Authors:** Fatma H. Shaker, Eman F. Sanad, Hesham Elghazaly, Shih-Min Hsia, Nadia M. Hamdy

**Affiliations:** 1 Department of Biochemistry, Faculty of Pharmacy, Ain Shams University, Cairo, Abassia, 11566 Egypt; 2 Department of Clinical Oncology, Faculty of Medicine, Ain Shams University, Cairo, Abassia, 11566 Egypt; 3 School of Food and Safety, Nutrition Research Center, Taipei Medical University Hospital, Taipei Medical University, Taipei, 110301 Taiwan; 4 Graduate Institute of Metabolism and Obesity Sciences, College of Nutrition, Taipei Medical University, Taipei, 110301 Taiwan

**Keywords:** ncRNA, piRNA, piR-823, Cancer hallmarks, Precision, In silico

## Abstract

PIWI-interacting RNAs (piRNAs) have received a lot of attention for their functions in cancer research. This class of short non-coding RNAs (ncRNA) has roles in genomic stability, chromatin remodeling, messenger RNA (mRNA) integrity, and genome structure. We summarized the mechanisms underlying the biogenesis and regulatory molecular functions of piRNAs. Among all piRNAs studied in cancer, this review offers a comprehensive analysis of the emerging roles of piR-823 in various types of cancer, including colorectal, gastric, liver, breast, and renal cancers, as well as multiple myeloma. piR-823 has emerged as a crucial modulator of various cancer hallmarks through regulating multiple pathways. In the current review, we analyzed several databases and conducted an extensive literature search to explore the influence of piR-823 in carcinogenesis in addition to describing the potential application of piR-823 as prognostic and diagnostic markers as well as the therapeutic potential toward ncRNA precision.

## Introduction

PIWI-interacting RNAs (piRNAs) are a newly discovered class of short non-coding RNAs (ncRNAs) in germ and somatic cells that consist of 26–30 nucleotides (nt) with a uridine base at the 5′-terminal or an adenosine base at the tenth position and a 2′-O-methyl at the 3′-terminal, that seem to have distinctive properties of all piRNAs (Aravin et al. 2006). These short chains of RNA were identified in germline cells and recognized as important modulators of germline maintenance (Huang et al. 2013a). Currently, piRNAs have a crucial role in controlling genomic expression in various disorders, including cancer, via several mechanisms such as transposon silencing, epigenetic modifications, and chromatin remodeling (Jia et al. 2022). The family of Argonaute proteins, divided into the Ago and PIWI subfamilies, is essential to short RNA-mediated gene regulatory pathways. Argonaute 1 (Ago1) and 2 (Ago2) members of the Ago subfamily of Argonaute proteins are known to be involved in small interfering RNA (siRNA)-mediated messenger RNA (mRNA) degradation and microRNA (miRNA)-mediated gene regulation, respectively (Yang et al. 2007). In *Drosophila,* the subfamily of PIWI proteins consists of Aubergine (Aub), Piwi, and Ago3, which are involved in retrotransposon silencing as well as germline stem cells’ maintenance and self-renewal. Ago3 and Aub are expressed in the cytosolic portion of germ cells, but Piwi is the only one of the three PIWI proteins, which is predominantly nuclear in germline and somatic cells present in the gonad and regulates piRNA synthesis in both types of cells (Huang et al. 2014a). Four PIWI proteins are expressed in humans, which are PIWI-like protein 1–4 (PIWIL1-4) (Tan et al. 2015). PIWI proteins influence transposon silencing, genome rearrangement, and epigenetics by creating complexes with piRNAs that control methylation and subsequently transposable elements (TEs) inhibition. Several types of cancer are either inhibited or promoted by transposon silencing and many other associated phenomena, including epigenetic modifications (Cheng et al. 2019). Although significant progress has been made in cancer research concerning diagnostics and therapeutics, the complete remission and survival of cancer patients remains a significant challenge. There is mounting evidence connecting piRNA/PIWI to cancer prognosis and carcinogenesis (Liu et al. 2019).

### Search strategy

A manual online search into two medical e-databases PUBMED and google scholar for (“piR-823” OR “piRNA-823”) AND (“piR-823 in Cancer”) AND (“piRNA in Cancer”) AND (“in silico”) AND (“Cancer hallmarks”) AND (“SNPs”) AND (“Piwi proteins”) AND (“Prognostic marker”) AND (“Diagnostic marker”) AND (“Nanoparticles”) AND (“Exosomes”) was done on June, 2023. Priority was given to meta-analysis, randomized clinical studies, systematic review, original papers, and narrative reviews, since, but not limited to, 2010.

## piRNA biogenesis

The way that piRNAs differ from miRNAs is that the piRNA precursors are often single-stranded and created from particular genome sites that include repeated motifs, and their coordinated signaling pathway is not dependent on dicer (Huang et al. 2014b). According to their origin, piRNAs are grouped into three types: (1) repeat-associated piRNAs, also known as “transposon-derived piRNAs,” from intergenic loci known as piRNA clusters and are rich in transposon segments, (2) mRNA-derived piRNAs produced from the 3′ UTR of mRNAs, and (3) long non-coding RNA (lncRNA)-derived piRNAs (Han and Zamore 2014). Transposon-derived piRNAs have a considerably better understanding of their biogenesis and functions than the other two types (Moyano and Stefani 2015). Following transcription, primary synthesis and “ping-pong” amplification are the main processes that transform piRNA primary transcripts into mature piRNAs (Jia et al. 2022).

The primary synthesis is based on the RNA polymerase II-mediated transcription of short sequences from piRNA precursors known as clusters. These transcripts are transported to the cytoplasm, where zucchini (Zuc) and its cofactor protein minotaur (Mino) trim them into smaller sequences to generate piRNA intermediates (Jia et al. 2022). The PIWI protein then binds to piRNA intermediates to form the PIWI/piRNA complex, which returns to the nucleus to initiate transcription-silencing mechanisms to inhibit the expression of its target genes by pairing the complementary bases of piRNAs and DNA (Czech and Hannon 2016).

The “ping-pong” amplification process produces a huge number of piRNAs in the cytoplasm. Through this process, piRNAs generate Ago3/piRNA or Aub/piRNA complexes by binding to Ago3 or Aub proteins instead of PIWI proteins (Xiol et al. 2014). The sequences in these complexes complement each other. In this way, an Ago3/piRNA complex identifies and trims an RNA sequence, producing a new RNA sequence that serves as a substrate for the formation of a new piRNA able to bind an Aub protein. Similarly, the resulting Aub/piRNA complex cuts a complementary RNA sequence, generating more RNA molecules, which in turn produce new Ago3/pirn complexes (Jia et al. 2022). As a result, the piRNA cytoplasmic product acts as a substrate for another piRNA molecule in an amplification manner. Finally, these piRNAs bind to PIWI proteins and return to the nucleus, where they inhibit the transcription of the targeted genes (Assumpção et al. 2015).

## piRNA regulatory functions

**Fig. 1:**
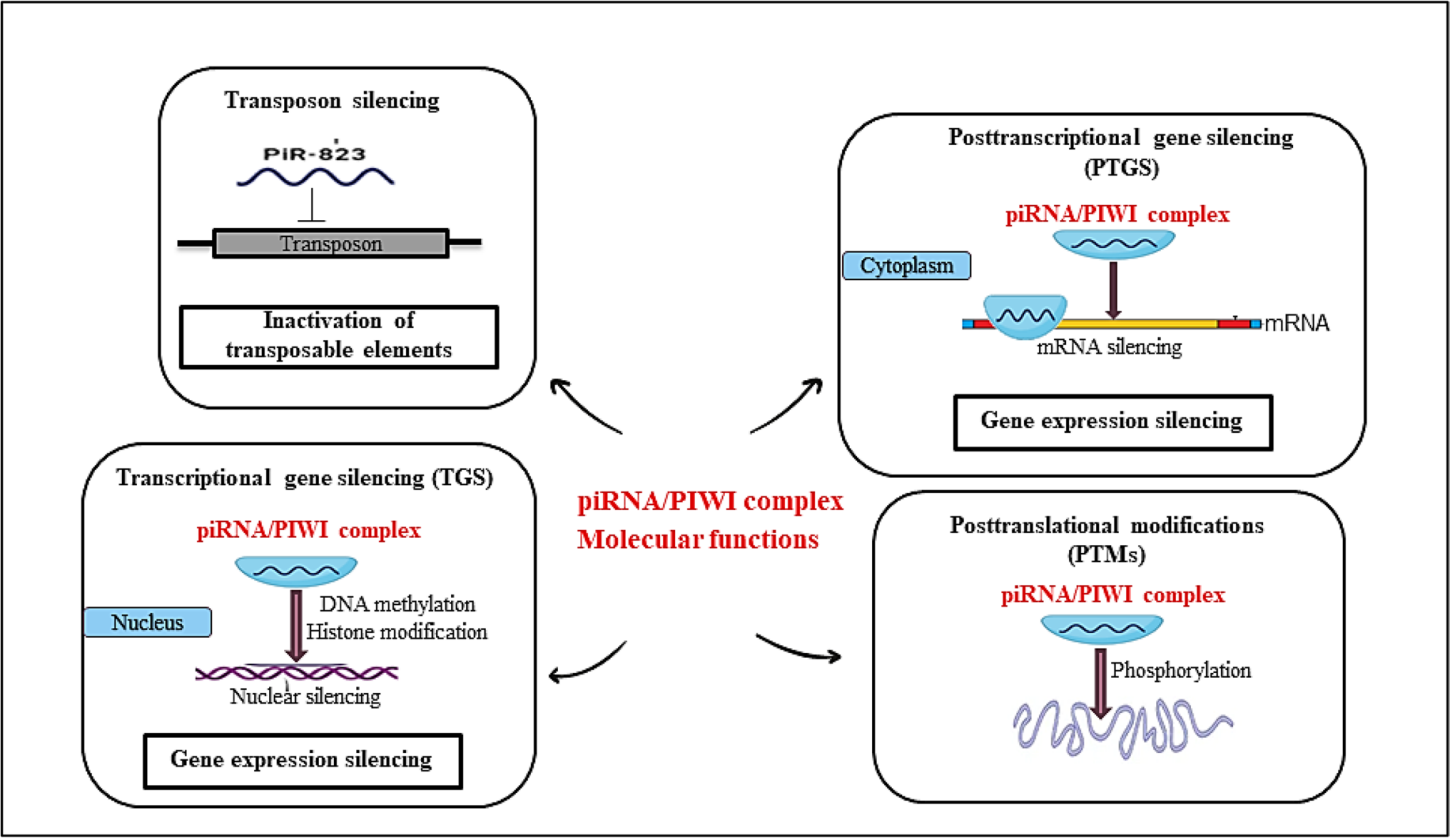
Regulatory molecular functions of piRNA/PIWI complex. This complex is involved in gene regulation through TEs inactivation, transcriptional gene silencing, posttranscriptional gene silencing, and posttranslational modifications

It has become evident that piRNAs are considered transcriptional regulation molecules through transposon silencing in addition to gene and protein regulation (Fig. 1).

### Transcriptional gene silencing (TGS)

The piRNA/PIWI complex exploits histone modifications and DNA methylation to control to transcriptional gene silencing (TGS). The piRNA/PIWI complex, after being produced in the cytoplasm, moves into the nucleus to combine with the genomic target to recruit silencing molecules and trigger the beginning of the TGS. DNA methyl transferase (DNMT) is recruited by the piRNA/PIWI complex to methylate sites containing CpG and alter the transcription function (Kuramochi-Miyagawa et al. 2008). Likewise, piR-30473 is implicated in the carcinogenesis of diffuse large B-cell lymphoma by modulating methylation of m6A RNA (Han et al. 2021). In primary multiple myeloma (MM) cells, de novo DNMT3A and DNMT3B were directly recruited by piR-823 to initiate overall DNA methylation (Yan et al. 2015).

### Posttranscriptional gene silencing (PTGS)

piRNAs are currently known to engage in post-transcriptional functions resembling miRNAs by acting on other cytoplasmic RNAs such as mRNA, transcribed pseudogenes, and lncRNA (Liu et al. 2019). For example, piR-55490 could bind to the mammalian target of rapamycin (mTOR), causing mRNA damage and thereby preventing lung cancer incidence and progression (Peng et al. 2016). Also, piR-1245 promotes tumor progression by binding to a class of downstream target genes that control cell survival, resulting in RNA silencing via aberrant base-pairing (Weng et al. 2018). Likewise, piR-823 binds to eukaryotic initiation factor 3 B (EIF3B) and upregulates the expression of transforming growth factor-1beta (TGF-1β) to initiate hepatic stellate cells during hepatic fibrosis (Tang et al. 2018).

### Posttranslational modifications (PTMs)

Post-translational modifications occur in the cytoplasm, where the piRNA/PIWI complex interacts with various transcription factors and controls their post-translational alterations, such as phosphorylation. In colorectal cancer (CRC), piR-823 promotes tumorigenesis by increasing the activation and transcriptional function of heat shock factor 1 (HSF1) (Yin et al. 2017). Moreover, the piR-54265/PIWIL2 complex interacts with the signal transducer and activator of transcription 3 (STAT3) to produce a complex that activates the phosphorylation of STAT3, thereby promoting CRC cell proliferation, metastasis, and chemoresistance (Mai et al. 2018).

### Transposone silencing

Transposons are mobile DNA components that account for less than 2% of the individual genome and utilize the genome and replicative machinery of the host cells for their existence and proliferation (Yuan et al. 2021). TEs can introduce new genetic material into the genome and frequently induce DNA modifications such as deletion, duplication, or inversion (Cheng et al. 2019). piRNAs preserve genomic integrity and stability through transposon silencing by forming an RNA-induced silencing complex (RISC). piRNAs bind to a certain transposon sequence through base complementarity. This mechanism effectively inhibits genomic transposition elements, preventing the cellular genes from being destroyed (Yuan et al. 2021). Furthermore, RISC can bind to PIWI proteins and target transposon transcripts in order to silence them and utilize active transposon transcripts as precursors for piRNA amplification through the ping-pong mechanism (Moyano and Stefani 2015). Surprisingly, new research has revealed that transposons, via the PIWI-piRNA pathway, actively control mRNAs and lncRNAs expression. Furthermore, pseudogenes control the expression of their related mRNAs via the piRNA/PIWI pathway (Wang and Lin 2021). According to a previous study, transposon regions in lncRNAs act as the targets for piRNAs formed from transposon-derived lncRNAs, which mark these lncRNAs for decay. A global analysis of the transposon dispersion in the human genome revealed that 83.4% of lncRNAs overlap with the minimum of one transposon, compared to only 6.2% of protein-coding sequences (Kelley and Rinn 2012). Numerous organisms have shown piRNAs to be involved in the transposon control of lncRNAs. The overexpression of transposon-containing lncRNAs in Miwi mutant mouse spermatocytes suggests that the piRNA pathway is the mechanism by which transposon regulates lncRNA expression (Vourekas et al. 2016).

## piRNA datasets and target predictions

### piRNA–mRNA and piRNA–lncRNA interactions

**Fig. 2:**
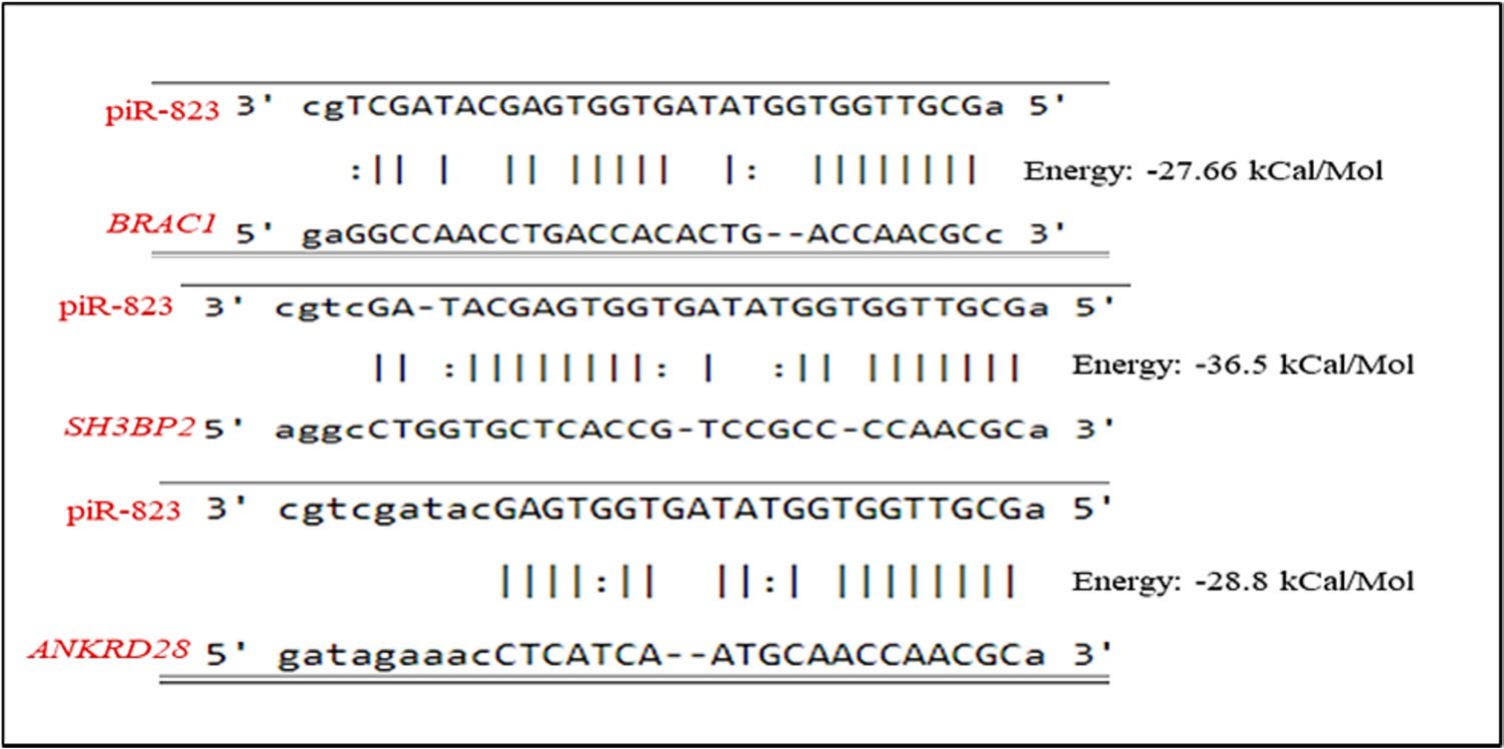
Predicted piR-823 target pairing in lncRNA for *BRAC1*, *SH3BP2*, and *ANKRD28* using piRNAQuest database (Accessed January, 2024)

The piRNA-mediated cleavage of TEs is a well-known function of piRNA. Nevertheless, recent studies have revealed the involvement of piRNAs in controlling the expression of mRNAs and lncRNAs. The cleavage of piRNA-targeted mRNA occurs at the tenth position from the 5′ terminal of the targeted piRNAs, which are antisense to the mRNA sequence (Wang and Lin 2021). piRNAs may be able to silence an mRNA by targeting its protein-coding sequence (CDS). For instance, Aub directly and piRNA-dependently targets the 3′ UTR and CDS of maternal mRNAs in the Drosophila to promote mRNA degradation (Barckmann et al. 2015). The incorporation of transposon sequences into mRNAs as piRNA targets involves dynamic mechanisms regulating mRNA expression, at least for certain cell types (Watanabe et al. 2015). piRNA-mRNAs interactions involve canonical mRNA decay machinery. Therefore, piRNA-mediated mRNA degradation also involves contemporaneous deadenylation and decapping of mRNAs in addition to PIWI slicing of the target mRNAs (Wang and Lin 2021). The PIWI/piRNA complex trims targeted lncRNAs like how it slices mRNAs. Then, mature piRNAs are integrated into PIWI proteins, which enable the PIWI complex to destroy target lncRNAs by targeting those lncRNAs with complemented transposon sequences (Sytnikova et al. 2014). piRNAQuest (http://dibresources.jcbose.ac.in/zhumur/pirnaquest2/start.php), a comprehensive database that provides information on piRNA features and piRNA expression in relation to various tissues. It offers a diverse narrative focusing on pseudogenes and predicts piRNA–mRNA and piRNA–lncRNA interactions and overlaps in various diseases and cancer. For instance, it showed target pairing between piR-823 and lncRNA for the *BRAC1* gene in breast cancer (BC), *SH3BP2* in renal cell carcinoma (RCC), and *ANKRD28* in hepatocellular carcinoma (HCC) (Fig. 2).

pirnaPre (http://www.regulatoryrna.org/software/piRNA/piRNA_target_mRNA/index.php) and pirScan (http://cosbi4.ee.ncku.edu.tw/pirScan/), tools designed for identifying and predicting piRNA targets. IsopiRBank (http://mcg.ustc.edu.cn/bsc/isopir/index.html) is a comprehensive online tool that offers piRNA isoform analysis, enrichment analysis, and target prediction. Moreover, piRNA targets and functions can be explained by the Gene Ontology (GO) and Kyoto Encyclopedia of Genes and Genomes (KEGG) pathway enrichment analyses of piRNA targets (Zuo et al. 2019). piRBase (http://bigdata.ibp.ac.cn/piRBase/) is another prediction tool for piRNA targets in lncRNAs in mice. There are several tools available for predicting miRNA targets; however, there are not many for piRNA targets. Research on prediction algorithms and tools for piRNAs and their targets is still in the early stages. Several tools, databases, and algorithms are dedicated to the detection and analysis of piRNA as collected in Table 1. Although piRNA bioinformatics research has evolved, further studies are required to overcome the limitations of the computational tools of piRNA (Liu et al. 2021). This is accomplished first by strengthening the development of piRNA databases since different databases have different names for piRNAs, making piRNA searches more difficult. Specifically, piR-823, one of the most studied piRNAs, has different aliases (piR-31143, piR-1282, piR-1119, piR-000823, piR-30928) stored in different databases such NCBI, piRNABank, piRBase, piRNAQuest, and piRNAdb. Secondly, since many databases, including pirScan and piRBase, lack data on human species, computational biologists should investigate piRNA to increase the diversity of species studied. Finally, piRNA-target prediction and disease association data should be improved to facilitate computational and experimental research.

**Table 1:** Details of databases for piRNAs identification and functional predictions

Database	Description	Species	Ref
ncRPheno	Identification and validation of ncRNAs associated with diseases	11 species	(Zhang et al. 2020)
ncRNAV	Identification of human diseases-related ncRNA variants	H	(Zhang et al. 2021)
piRSNP	A platform for identifying piRNA-related variants and their roles on piRNA functions in cancer	H and M	(Liu et al. 2023)
piRNA-eQTL	Demonstrates the effects of piRNA-related variants expression using TCGA data and is an easy-to-use platform for the analysis of the quantitative expression of cis-piRNA trait locus (eQTLs) for 33 cancer types	H	(Xin et al. 2021)
piRPheno	Manually curated platform that demonstrates experimentally evidence based-associations between piRNAs and relevant disease phenotypes	H	(Anon n.d.-b)
piRDisease v1.0:	Manually curated platform for piRNA-related diseases which provides comprehensive details about the piRNA in the relevant disease, supported by experimental evidence, a succinct description, sequencing information, and location data	H, M, R	(Muhammad et al. 2019)
IsopiRBank	A comprehensive resource of piRNA isoforms target prediction and enrichment analysis	D, H, M, Danio rerio	(Zhang et al. 2018)
PiRBase V3.0	Manually curated database of piRNAs sets, piRNA clusters, piRNAs variants, and piRNA expression data. It also provides information of piRNA function analyses, piRNA annotation, and splicing-junction piRNA	21 species	(Wang et al. 2022a)
piRNABank	A very user-friendly tool that provides relevant data on piRNAs as well as sequences that have been empirically determined. Each piRNA or piRNA cluster is also shown on a graphical genome-wide map	D, H, M, R, Ornithorhynchus anatinus	(Xu et al. 2008)
PirnaQuest	A comprehensive database that offers piRNA annotation according to their chromosomal locations in gene, lncRNA, and syntenic regions. It also includes details on every potential piRNA cluster and important patterns found in the piRNAs that make up a cluster	25 species	(Sarkar et al. 2014)
piRNAdb	Sequence storage and search system, including datasets, alignments, clusters, and targets	C, D, H, M, R, Cricetulus griseus	(Anon n.d.-a)
piRTarBase	Platform determines expected mRNA targets of particular piRNAs and explores piRNA-targeting sites, regulatory impacts on endogenous genes	C, Caenorhabditis briggsa	(Wu et al. 2019)
piRNAtarget	Integrated database to investigate the functions played by piRNA and its targets in addition to annotations, sequences, mutation, and methylation patterns in human piRNAs	H	(Jiang et al. 2016)
pirScan	Anticipate piRNA-targeting sites and prevent transgenes from being persistently germline silenced, which has rendered many constructions useless	C. elegans, C. briggsae	(Wu et al. 2018)
miRanda	miRNA-target prediction software is used to predict piRNA targets because of similarity patterns of degradation/regulation between both	H	(Liu et al. 2018b)

*C*, Caenorhabditis elegans; *D*, Drosophila melanogaster; *H*, Homo sapiens; *M*, Mus musculus; *R*, Rattus norvegicus; *SNPs*, single-nucleotide polymorphisms; TCGA, the cancer genome atlas

### In silico identification and expression of piR-823

**Fig. 3:**
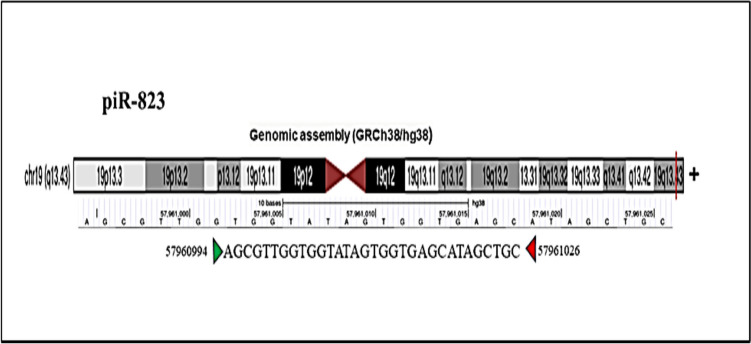
Illustration of genomic localization of piR-823 using piRNAdb database (https://www.pirnadb.org/information/pirna/hsa-piR-1119) (Accessed May,2024)

**Fig. 4:**
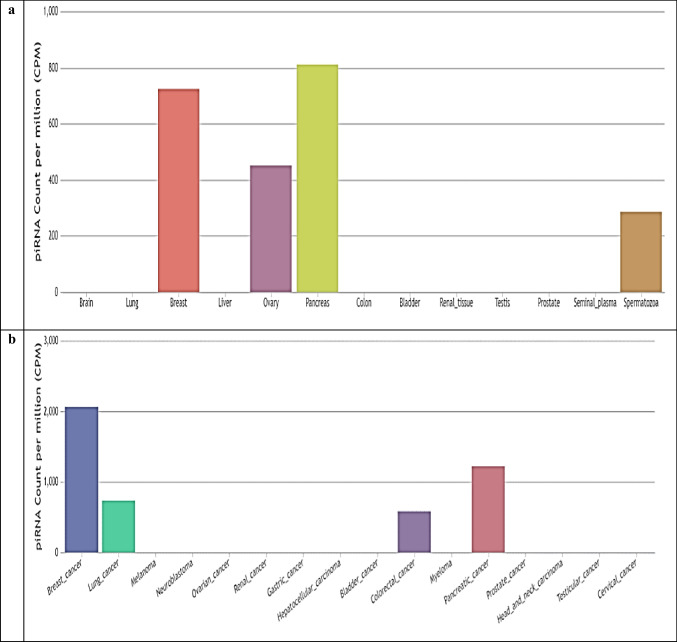
Expression level of piR-823 (piRNA-30928) in **a** different tissues. The expression graph of piR-823 is represented in count per million (CPM). As shown, the highest expression of piR-823 was found in pancreatic tissues using piRNAQuest V.2 database http://dibresources.jcbose.ac.in/zhumur/pirnaquest2/expressn_view.php?organism=hsa&pirna_id=hsa_piRNA_30928&submit=Submit. **b** Different cancers. The expression graph of piR-823 is represented in count per million (CPM). As shown, the highest expression of piR-823 was found in breast cancer using piRNAQuest V2 http://dibresources.jcbose.ac.in/zhumur/pirnaquest2/expressn_view.php?organism=cancer&pirna_id=hsa_piRNA_30928&submit=Submit (Accessed May, 2024)

**Fig. 5:**
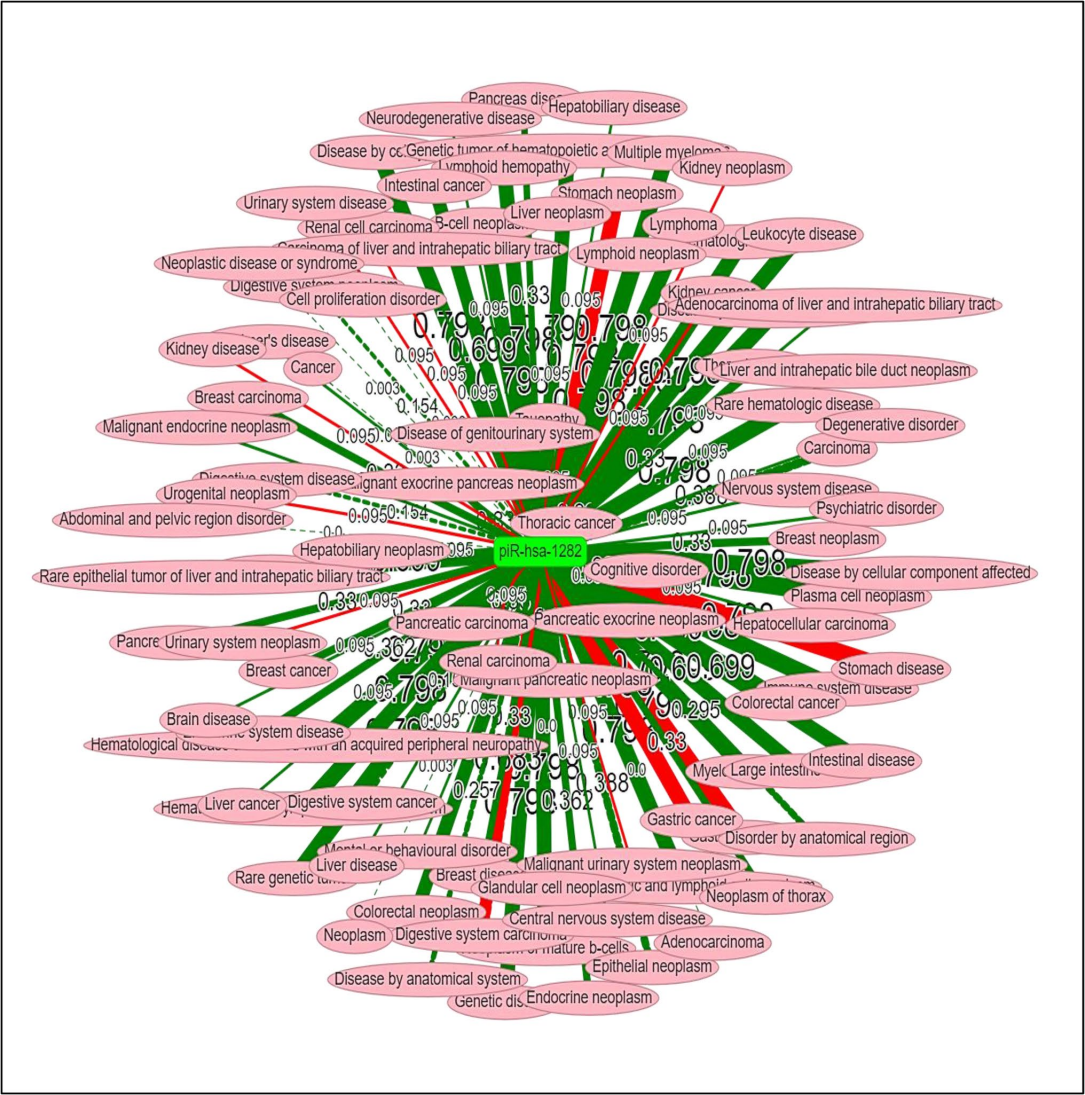
piR-823 expression in different human cancer using PiRpheno V2.0 (http://www.biomedical-web.com/pirpheno/Networklyzer.jsp) (Accessed May, 2024). The lines that are thicker signify a higher confidence level (ranging from 0 to 1) for the piRNA-disease relationship; whereas, the green lines show a negative correlation between the piRNAs and the illness phenotypes. The red lines show a positive correlation between the two. The piRNA-disease relationships with contradicting evidence are shown by the dashed lines

Abnormal expression of piR-823 has been associated with several tumors and may have an oncogenic or tumor suppressor effect on the onset, progression, and spread of cancer. From the piRNAdb database (https://www.pirnadb.org/index) and UCSC Genome Browser (https://genome.ucsc.edu/index.html), piR-823 contains 32 bases located in chromosome 19 (Fig. 3). Using piRNAQuest V2., piR-823 is highly expressed in different tissues as shown in Fig. 4a and in different cancers as shown in Fig. 4b. Moreover, PiRpheno V2.0 database (http://www.biomedical-web.com/pirpheno/pirpheno.html) showed the expression profile of piR-823 in various diseases and cancer as shown in Fig. 5. piR-823 was found to be associated either positively or negatively with 92 different diseases and cancers. Hematopoietic, lymphoid, and myeloma neoplasm were among the highest confidence score correlation. Interestingly, piR-823 was found to be associated with neurodegenerative diseases such as Alzheimer’s disease.

## piR-823 interactions in different cancer types

Though the potential role of piR-823 in cancer is being highly investigated, its functional role in human cancer is poorly known. The expression and function of piR-823 in various human cancers have been summarized, as shown in Tables 2 and 3.

**Table 2:** Upregulated expression and clinical utility of piR-823 in different human cancers

Cancer type	Method of detection	Gene/pathway	Clinical utility	Samples tested	Mechanism/study outcome	Ref
Colorectal cancer	PCR	HSF1, HSP	Therapeutic	Patients’ tissues, cell lines (HCT116/DLD‐1/FHC)	piR-823 increases phosphorylation, transcriptional activity of HSF1	(Yin et al. 2017)
	PCR	PINK1, parkin, MFN2	Therapeutic	Patients’ tissues, cell lines (HCT116/DLD‐1/FHC)	Induce PINK1degradation, inhibiting mitophagy, apoptosis in CRC cell lines	(Wang et al. 2022b)
	PCR microarray	G6PD/HIF‐1α pathway	Therapeutic	Patients’ tissues, cell lines (HCT‐116/Lovo/293 T)	Promotes proliferation, invasion, apoptosis resistance of CRC cells via modulating G6PD/HIF‐1α pathway	(Feng et al. 2020)
	PCR	–	Diagnostic	Patients’ serum	An effective addition to CEA for CRC clinical diagnosis	(Tong et al. 2023)
	PCR	–	Diagnostic	Patients’ serum	Efficient biomarker for CRC diagnosis detected by biosensor	(Sun et al. 2022)
	PCR	–	Diagnostic	Patients’ serum, tissues	Screening non-invasive method in CRC, elevated in serum and tissues	(Sabbah et al. 2021)
Breast cancer	PCR	DNMT1, DNMT3A/B, WNT	Therapeutic	Patients’ tissues, animals, cell lines (MCF-7/T47D/ALDH + CSC)	Inducing stemness in luminal BC, regulating DNMTs, WNT pathways expression	(Ding et al. 2021)
	PCR	hTERT, PI3K/AKT/mTOR	Therapeutic	Cell lines (MDA-MB-231)	Control ERα expression, hTERT, PI3K/AKT/mTOR expression pathway, impact TNBC cell proliferation and cellular properties	(oner 2022)
	PCR	Estrogen, androgen	Therapeutic	Cell lines (MCF-7, MDA-MB-231)	piR-823 expression modulated by estrogen and androgen	(Öner et al. 2016)
Multiple myeloma	PCR microarray	VEGF, IL-6, ICAM-1, CXCR4, Bax, Bcl2	Prognostic, therapeutic	Patients’ tissues, cell lines (RPMI8226/ARH-77/U266)	Enhanced expression of VEGF, IL-6, ICAM-1, CXCR4, and Bcl2, reducing Bax to attenuate apoptosis, increase ROS generation and tumor invasion	(Yan et al. 2015)
	PCR	DNMT3B	Therapeutic, prognostic	Patients’ blood, animal, cell lines (RPMI8226/NCI-H929)	MDSCs activate DNMT3B, upregulate piR-823 expression	(Ai et al. 2019)
	PCR	DNMT3A, DNMT3B, p16^INK4A^	Therapeutic	Patients’ serum, BM, cell lines (ARH-77/EA. hy926/RPMI 8226)	de novo DNA methylation, DNMT3A and 3B, represses methylation-silenced tumor inhibitor, p16^INK4A^	(Li et al. 2019)
Hepato-cellular carcinoma	PCR	hTERT, HIF-1α, MMP-2, MMP-9	Therapeutic	Cell lines (HepG2)	Promotes adhesion, telomerase activity, HepG2 proliferation	(oner 2021)
	PCR	EIF3B, TGF-1β, α-SMA, COL1a1	Therapeutic	Cell lines (HSCs), animal	piR-823 upregulation, promote HSCs proliferation, hepatic fibrosis	(Tang et al. 2018)
	Small RNA sequencing	-	Diagnostic	Patients’ tissue	piR-823 levels increased gradually from cirrhosis to dysplastic nodules	(Rizzo et al. 2016)
	PCR	MMP-2, MMP-9	Therapeutic	Cell lines (HepG2)	piR-823 expression affected adhesion, viability, HepG2 proliferation	(Öner et al. 2022)
Prostate cancer	PCR	Estrogen, androgen	Therapeutic	Cell lines (LNCaP, PC-3)	piR823 expression regulated by estrogen and androgen	(Öner et al. 2016)
Esophageal cancer	PCR	DNMT3B	Therapeutic, diagnostic, prognostic	Patients’ tissues	Abnormal DNA methylation via DNMT3B and tumor pathogenesis	(Su et al. 2020)

*DNMT*, DNA methyl transferase; *EIF3B*, eukaryotic initiation factor 3 B *TGF-1β*, transforming growth factor-1beta, *α-SMA*, alpha-smooth muscle actin; *COL1a1*, collagen type 1 alpha 1; *hTERT*, human telomerase reverse transcriptase; *HSF*, heat shock factor; *PI3K*, phosphatidylinositol 3-kinase; *AKT*, protein kinase B; *mTOR*, mammalian target of rapamycin; *HCC*, hepatocellular carcinoma; *MMP*, matrix metallopeptidase; *VEGF*, vascular endothelial growth factor; *IL-6*, interleukin-6; *ICAM-1*, intercellular adhesion molecule, *CXCR4*, C-X-C motif chemokine receptor 4; *Bax*, Bcl-2-associated X protein; *Bcl2*, B-cell lymphoma; *ROS*, reactive oxygen species; *WNT*, wingless-related integration site; *HSF1*, heat shock factor 1; *HSP*, heat shock protein; *PINK1*, PTEN-induced kinase1; *MFN2*, mitofusin2; *G6PD*, glucose-6-phosphate dehydrogenase; *HIF‐1*, hypoxia inducible factor-1; *CRC*, colorectal cancer; *CEA*, carcinoembryonic antigen; *MM*, multiple myeloma; *BC*, breast cancer; *BM*, bone marrow; *CSC*, cancer stem cell; *MDSCs*, myeloid-derived suppressor cells; *HSCs*, hepatic stellate cells; *PCR*, polymerase chain reaction

**Table 3:** Downregulated expression and clinical utility of piR-823 in different human cancer

Cancer type	Method of detection	Clinical utility	Samples tested	Mechanism/study outcome	Ref
Gastric cancer	PCR	Diagnostic	Patients’ blood	Positively associated with TNM and distant metastasis	(Cui et al. 2011)
	PCR, microarray	Therapeutic	Patients’ tissues, GES-1/MGC-803/SGC-7901 cells	Inhibited cell proliferation in vitro and in vivo	(Cheng et al. 2012)
Renal cell carcinoma	PCR	Diagnostic, prognostic	Patients’ tissue, serum, urine	Positively correlated with poor prognosis	(ILIEV et al. 2016)
	PCR	Diagnostic, prognostic	Patients’ tissues	Significant association of PIWIL1, PIWIL2, PIWIL4 expression with RCC patients’ survival	(Slaby et al. 2016)

*TNM*, tumor node metastasis; *PIWIL*, PIWI-like proteins; *RCC*, renal cell carcinoma

### Colorectal cancer

As the most common form of gastrointestinal (GI) cancer, colorectal cancer (CRC) is now the third most common cause of cancer-related death. Owing to the diverse nature of CRC, several pathways, such as chromosomal instability, microsatellite instability, epigenetic alterations (Abd El Fattah et al. 2023; El-Sheikh et al. 2023; Emam et al. 2022; Rizk et al. 2024), and abnormal expression of tumor oncogenes or suppressor genes, can influence CRC initiation and proliferation (Ameli Mojarad et al. 2022). However, the majority of patients are diagnosed in late stages due to insufficient prognostic and diagnostic biomarkers, reducing their chances of receiving early treatment; thus, identifying robust and precise therapeutic indicators is essential for managing CRC (Mojarad and Mojarad 2021). piRNAs, through their binding to PIWI, have a vital role in the pathogenesis of CRC, and genetic variants in piRNAs may modulate CRC susceptibility (Henaoui et al. 2017). Cancer research emphasizes finding novel, reliable diagnostics for the early detection of GI malignancies and therapeutic targets that function as targeted treatments to lower mortality. Consequently, studies have demonstrated that piRNAs have important clinical implications as CRC therapeutic targets and tools for diagnosis. Thirty-one deregulated piRNAs, including piR-5937 and piR-28876, showed adequate diagnostic performance in large-scale piRNA expression analysis in serum samples from colon cancer patients compared to normal groups, implying the clinical utility of piRNAs as early diagnostic markers for differentiating between cancer and normal individuals (Vychytilova-Faltejskova et al. 2018). piR-823 is one of the piRNAs that contributed to the tumorigenesis of CRC and could be utilized as a possible CRC therapeutic target. Upregulation of piR-823 promotes both proliferation and tumorigenesis in CRC tissues by upregulating the expression of HSF1, a potent carcinogenesis trigger, and by influencing the phosphorylation of STAT3 (Yin et al. 2017). In a different study, it was found that piR-823 targeted PTEN-induced kinase 1 (PINK1), promoting the proteasome-mediated degradation of PINK1 and preventing mitophagy and cell death in CRC cell lines. piR-823 reduced the ubiquitination of the mitochondrial proteins mitofusin (MFN2) and translocator of the outer mitochondrial membrane (TOM20), which may have contributed to inhibiting mitophagy mediated by PINK1/Parkin signaling (Wang et al. 2022b). Similarly, piR-823 increases glucose-6-phosphate dehydrogenase (G6PD) expression, which in turn increases the consumption of glucose by cancer cells while decreasing the amount of intracellular reactive oxygen species (ROS). This suppresses the ubiquitination of hypoxia-inducible factor 1 alpha (HIF-1 α). Therefore, via modifying the G6PD/HIF-1α pathway, piR-823 increases CRC cell growth, invasion, and resistance to apoptosis (Feng et al. 2020). Because of the overexpression of piR-823 in CRC patients’ serum and tissues, it holds promise as a convenient, non-invasive screening technique for CRC (Sabbah et al. 2021). piR-823 can be utilized for clinical diagnosis of CRC in addition to carcinoembryonic antigen (CEA) (Tong et al. 2023).

### Hepatocellular carcinoma

Hepatocellular carcinoma (HCC) is the second most common cause of cancer-related deaths in both men and women. It develops as a consequence of a progressive process that frequently starts with the morphologically identifiable low- and high-grade dysplastic nodules within a cirrhotic liver developing premalignant lesions. Numerous studies have identified and characterized many abnormal gene expressions, single-nucleotide polymorphisms (SNPs), and noncoding RNAs (ncRNAs) in HCC (Abaza et al. 2023; El-Aziz et al. 2023; El-Derany et al. 2016; Hammad et al. 2023). The multifactorial characteristics of the pathways, which emphasize the importance of epigenetic variables including miRs, lncRNAs, and piRNAs, may help to understand the pathogenesis of HCC (Eldosoky et al. 2023). piR-823 is a novel player in the molecular processes that lead to hepatocarcinogenesis. Whereas, piR-823 levels increase steadily from cirrhosis to low-grade and then high-grade dysplastic nodules and HCC, suggesting that they could be new helpful biomarkers for differentiating dysplastic and neoplastic liver lesions (Rizzo et al. 2016). Moreover, piR-823 may influence liver fibrosis by activating hepatic stellate cells (HSCs). Overexpression of piR-823 increased HSCs viability and proliferation by increasing the generation of alpha-smooth muscle actin (α-SMA) and collagen type 1 alpha 1 (COL-1a1). piR-823 interacts with EIF3B, and this complex induces TGF-1β expression, activating dormant HSCs. Thus, inhibiting piR-823 and HSCs might represent a new strategy for treating liver fibrosis (Tang et al. 2018). Similarly, piR-823 might act as a therapeutic target in HCC. As a recent study revealed that 4-hydroxycoumarin treatment caused upregulation of piR-823, matrix metallopeptidases (MMP-2, and MMP-9) expression, which are important features of cellular survival mechanisms that led to the loss of viability, adhesion, and proliferation of HepG2 cells (Öner et al. 2022). Furthermore, 1,25-dihydroxy vitamin D therapy increased proliferation, adhesion, differentiation, and telomerase activity in HepG2 cells through increasing piR-823 expression while decreasing HIF-1α, MMP-2, and MMP-9 expression. Unfortunately, the active form of vitamin D may influence the epigenetic mechanisms of HCC cells via piR-823 expression and may not be beneficial for as an anti-cancer in HCC patients (oner 2021).

### Gastric cancer

With over one million new cases identified each year, gastric cancer (GC) is the fifth common cause of cancer-related deaths worldwide (Ameli Mojarad et al. 2022). The overall survival of people with GC has improved as early detection of cancer has increased, and radical surgery has become more widely used. However, the prognosis for advanced GC is still poor, and choices for safe and effective adjuvant therapy are limited (Li 2014). As a result, finding valuable early markers is critical for GC diagnosis and prognosis. The abnormal expression of piRNAs and PIWI proteins has been reported in GC tissues, implying their crucial role as a promising biomarker in gastric tumorigenesis. piR-823 expression was considerably lower in GC patients’ peripheral blood compared to healthy controls. With a correlation found between the levels of piR-823 and both distant metastasis and tumor node metastasis (TNM) indicating that piR-823 detection may serve as a prognostic marker for GC (Cui et al. 2011). Overexpression of piR-823 inhibited the growth of both GC cells and transplanted tumors, suggesting the involvement of piR-823 in the development of GC and implying that piR-823 may have a tumor suppressor effect on the GC progression (Cheng et al. 2012). Both piR-823 and piR-651 were more sensitive for GC than CEA and carbohydrate antigen 19–9 (CA19-9) (Cui et al. 2011). piRNAs are not easily degraded because they are short fragments, and the levels of piR-651 and piR-823 in blood samples are reasonably stable and may be detected and isolated from body fluids as they can cross easily through the cell membrane (Chalbatani et al. 2019). PIWIL1 expression has elevated in GC cells, indicating that a high level of PIWIL1 expression may be a crucial prognostic indicator for predicting the survival of GC patients. By modulating the expression of oncogenes and tumor suppressor genes, PIWIL1 may accelerate GC pathogenesis and progression (Guo et al. 2020). The precise mechanisms by which PIWIL1 and piR-823 contribute to the proliferation and metastasis of GC are still unclear. Nonetheless, favorable prospects are indicated by the aberrant expression of piRNA and PIWI proteins in GC tissues compared to normal tissues.

### Breast cancer

Breast cancer (BC) is one of the most common malignancies and the leading cause of cancer-related mortality in women worldwide. According to the expression analysis profiles of progesterone receptor (PR), estrogen receptor (ER), and human epidermal growth factor receptor 2 (HER2), BC is divided into four subtypes: luminal, HER positive, basal-like, and normal-like (Ding et al. 2021). Despite the significant improvements in human BC therapeutics, tumor recurrence and metastasis remain incurable, owing to the chemo-resistance from the ncRNA effect (Mahmoud et al. 2021b), as well as more tumor growth promotion from activated oncogenes vs tumor suppressor genes inhibition (Mahmoud et al. 2021a; Rezaeean et al. 2021; Salama et al. 2021). All these effects are regulated via ncRNAs and one of which to be explored and examined is piRNAs. According to a recent study, by methylating a particular gene, piRNAs can influence the proliferation, metastasis, cancer development, and prognosis of BC. This suggests that piRNAs may be a promising target for therapy and a novel class of biomarkers for the early detection and prognosis of BC (Öner et al. 2016). piR-823 is a novel modulator of cancer stem cells (CSCs) in luminal BC since suppression of piR-823 significantly reduced the growth of tumors and the proliferation of cancer cells both in vitro and in vivo by controlling DNMTs expression and by increasing the DNA methylation levels of tumor suppressor genes like adenomatous polyposis coli (APC), consequently, activating the WNT signaling pathway, which in turn induces cancer stemness in BC (Ding et al. 2021). piR-823 reacted to the hormonal therapy in a cancer subtype-specific way, as external treatment of estrogen increased the expression of piR-823 in the basal-like subtype triple-negative (ER, PR, and HER2 negative) (Öner et al. 2016). Meanwhile, decreasing the expression of piR-823 levels in the luminal subtype (ER and PR). As a result, high piR-823 expression promotes tumorigenesis in luminal BC, and piR-823 suppression may be used to predict patients’ therapeutic response to hormone therapy. A recent study revealed that inhibition of piR-823 increased ER-α expression while decreasing the expression of human telomerase reverse transcriptase (hTERT) and the phosphatidylinositol 3-kinase (PI3K)/AKT/mTOR pathway, which influences cell proliferation and cellular properties in triple-negative BC cells (oner 2022).

### Multiple myeloma

Multiple myeloma (MM) is a B-cell-derived cancer that causes clonal proliferation of plasma cells in the bone marrow (Khalili et al. 2023; Robak et al. 2018). Malignant plasma cell proliferation and MM development are associated with aberrations in the bone marrow microenvironment. Secondary leukemia and extramedullary disease might result from the separation of MM cells from the bone marrow microenvironment (Furukawa and Kikuchi 2015). CSCs persisting in the bone marrow of MM patients are one of the underlying mechanisms of drug resistance and relapse because these cells have an infinite potential for self-regeneration and drug resistance (Li et al. 2021). Another mechanism is genetic and epigenetic abnormalities, including DNA methylation and histone modifications, in various genes, particularly tumor suppressors (Robak et al. 2018). CSCs and myeloid-derived suppressor cells (MDSCs), major cellular factors in the tumor microenvironment in MM, can alter the tumor phenotype and influence patient prognosis and outcome. Granulocytic-MDSCs (G-MDSCs), a type of MDSC, increased the expression of piR-823, which in turn enhanced DNA methylation and promoted the tumorigenesis of MM cells (Ai et al. 2019). The ability of G-MDSCs to retain stemness diminished when silencing piR-823 in MM cells, which reduced tumor growth and angiogenesis in vivo, implying that piR-823 might be a unique anti-cancer therapy that targets both G-MDSCs and CSCs in the MM microenvironment (Ai et al. 2019). Endothelial cells (ECs) in the tumor microenvironment are highly required for the proliferation, survival, and dissemination of MM cells (Korn and Méndez-Ferrer 2017). Extracellular vehicles (EVs) derived from MM consist of functional proteins, mRNAs, miRNAs, and piRNAs (Raposo and Stoorvogel 2013). PiR-823 levels were significantly greater in peripheral EVs obtained from patients with MM (Li et al. 2019), suggesting a potential role for piR-823 in the diagnosis and prognosis of MM. piR-823 delivered to ECs by EVs derived from MM stimulates EC spread, angiogenesis, and proliferation which may eventually contribute to the progression of MM (Li et al. 2019). Another study reported that silencing piR-823 in MM cells reduced the release of vascular endothelial growth factor (VEGF), resulting in diminished angiogenic activity (Yan et al. 2015). All these results support the oncogenic involvement of piR-823 in the biology of MM and justify the need for exploring piRNA-targeted therapeutic approaches in MM.

### Genitourinary cancers

Studies on renal cancer have revealed a strong correlation between piRNA and the disease’s prognosis and metastasis. Additionally, in RCC, the most common kidney cancer in adults, the expression levels of PIWIL1, PIWIL2, and PIWIL4 gradually decrease with increasing clinical stage and a worsening prognosis (ILIEV et al. 2016). piR-823 has a complex function in the tumorigenesis and prognosis of RCC. Preliminary results show that piR-823 is highly expressed in urine and has a great diagnostic value in RCC patients (ILIEV et al. 2016). Another study reported the association of PIWIL1 in the piR-823-mediated pathogenesis of RCC through the deregulation of PIWIL1 and piR-823 in RCC tumor tissue with a positive correlation, as well as a substantial association has been observed between PIWIL1 and PIWIL2 and PIWIL4 expression and RCC survival (Slaby et al. 2016). Gender-specific steroid hormones are important in the development and pathophysiology of cancers of the reproductive system, such as androgen-dependent or androgen-independent prostate cancer in men (Feldman and Feldman 2001). piR-823 is one of the piRNAs important for cancers that are gender-dependent. Its overexpression in cancer is thought to be mediated by hormones (Öner et al. 2016). Increasing hormone levels, such as androgen, in prostate cancer cells, showed that the elevated piR-823 expression exhibited oncogenic properties in both androgen-dependent and androgen-independent prostate cancer cell lines and that this property increased as hormone levels increased (Öner et al. 2016).

### piR-823 SNPs in cancer

While most of the research has been on the aberrant epigenetics and gene expression of piRNAs in cancer, a few studies have also documented the relation between piRNAs and SNPs along with other genetic variations. Finding SNPs in particular or PIWI proteins was linked to a patient’s risk of developing cancer (Yao et al. 2022). Seven SNPs in nine piRNAs were evaluated for CRC susceptibility, and SNP rs11776042 in piR-015551 was found to be substantially linked to a lower risk of CRC. Results suggested that the rs11776042 thymine to cytosine (T > C) substitution affected the energy of the piRNA secondary structure and that might influence the role of piRNA on CRC development (Chu et al. 2015). Genotypic analysis of several SNP-containing piRNAs indicated a strong association between a higher risk for developing BC and SNP rs1326306 G > T in piR-021285 (Fu et al. 2015). A post-genome-wide association study (GWAS) and functional analyses of piRNAs in glioma-genesis revealed that SNP rs147061479 in piR-598 enhances the incidence of gliomas by eliminating piR-598 tumor-suppressive function (Jacobs et al. 2016). Another substantial evidence supporting the potential role of piRNA variants in lung cancer was found in a different post-GWAS study that identified SNP (rs1169347) as strongly related to lung cancer risk, and it can be matched into piR-5247 and piR-5671 (Ye 2016). Compared to piRNAs, SNPs in PIWI and piRNA-PIWI pathway-related genes are more prevalent and significantly linked to cancer risk (Lin et al. 2019; Roy et al. 2020; Zhang et al. 2016). SNPs identified specifically in piR-823, however, have not yet been reported, which calls for more thorough molecular research and genotyping screening. Exploring the association between ncRNAs-SNPS, and more precisely, piR-823 SNPs, and the cancer mechanism(s), is important to investigate to link certain polymorphisms to changes in cancer incidence, progression, or remission in the future. This represents a step toward the precision of ncRNA.^1^

Additionally, because piRNA variants have been experimentally verified as a novel class of biomarkers and therapeutic targets, it is now crucial to identify and understand the connections between piRNA variants and human diseases. Numerous computational databases and techniques have been developed to annotate piRNA variants due to their clinical and functional significance. For example, ncRNAVar (http://www.liwzlab.cn/ncrnavar/) offers association data between human diseases and validated ncRNA variants using computational annotation and manual curation of 2650 publications (Zhang et al. 2021). Also, piRNA-eQTL (http://njmu-edu.cn:3838/piRNA-eQTL/) provides analysis between SNPs and piRNA expression over 33 types of cancer using the cancer genome atlas (TCGA) program (Xin et al. 2021). Researchers may find it useful to use piRSNP (http://www.ibiomedical.net/piRSNP/) to thoroughly characterize SNPs associated with piRNA in humans and mice, as this information can be used to study piRNA functions in the future (Liu et al. 2023). These databases are a valuable tool for motivating researchers to investigate more ncRNA variants, which helps in understanding the roles that piRNAs and genetic variants play in cancer development.

## piR-823 clinical utility in cancer

### piR-823 as non-invasive cancer bio-molecular marker

piRNAs have received a lot of attention for their potential to be utilized in the diagnosis and prognosis of various types of cancers (oner 2022; Yan et al. 2015). Quantitative variations in the expression level of these piRNAs might help identify the origin of cancer tissue as a portion of piRNAs might be expressed in a way that is very cancer-specific (Cheng et al. 2011). A growing body of evidence revealed that piRNAs may be useful diagnostic and prognostic biomarkers in cancer, even though the proposed mechanisms need more investigation and may not fully explain piRNAs’ role as cancer biomarkers. piRNA expression in the body’s fluids, including blood, plasma, serum, saliva, sputum, and urine, is comparatively constant and readily detectable (Mei et al. 2013). This stability could be attributed to piRNAs’ 2′-O-methyl modification, which shields them from 3′ uridylation and truncation, two processes that affect the integrity of short RNAs (Jeong et al. 2021). Furthermore, piRNAs are just 26–32 nt long, a tiny RNA fragment that makes them more resistant to degradation than other large RNAs and allows them to easily cross various cell membranes. These properties indicate that piRNAs can be readily found and isolated from patient specimens (Chalbatani et al. 2019). As explained above, piR-823 overexpression in various cancers including colon, liver, renal, and breast cancer tissues compared to normal tissues, indicating that piR-823 may serve as a promising diagnostic biomarker for these cancers (Table 2). The prognosis of cancer has also demonstrated significant promise for piRNAs. Multiple studies revealed a correlation of piR-823 with TNM, distant metastasis, histopathology type, or cancer progression in several cancer types, implying that piR-823 could be an independent prognostic marker (Yan et al. 2015; Ai et al. 2019; Su et al. 2020; ILIEV et al. 2016). Another reason for utilizing piRNA as a biomarker is the encapsulation of certain piRNAs in the blood into exosomes and microvesicles, two types of extracellular vesicles, thereby protecting them from ribonuclease digestion. EV-piRNAs have shown promise as a diagnostic tool for cancer types (Goh et al. 2022). As identified, piR-823 aggregates in extracellular vesicles (EVs) from the peripheral blood of MM patients and cell lines. It is essential for the cellular interaction between the endothelial cells and myeloma cells in the tumor microenvironment. It is possible to successfully transfer EVs harboring piR-823 from MM cells to ECs and change their biological properties to establish an environment supportive of MM cells’ survival (Li et al. 2019). Collectively, aberrantly expressed piRNAs in human tumors represent a novel class of biomarkers for prognosis and diagnosis. Exosomes are one of the EVs produced from the multi-vesicular endosome pathway and are packed with biologically significant molecules like DNA, mRNA, and ncRNAs (Becker et al. 2016). Therefore, exosomes with a diameter ranging from 30 to 150 nm can serve as the best cargo for carrying information from cancer cells, and that information could be easily found in serum exosomes (Gu et al. 2020). Exosomes have gained researchers’ interest in cancer therapy because of their distinctive properties, including biocompatibility, minimal toxicity, circulatory stability, tumor specificity, and high tumor penetration capacity (Rizk et al. 2024). RNAs in plasma exosomes are differentially expressed and considered a promising biomarker for various cancer types; however, more research is needed to explore the novel content of exosomes especially piRNAs which account for 20–30% of total RNA (Ma et al. 2020). That suggests the great potential of exosomal piRNAs as an early non-invasive diagnostic and prognostic tool in cancer patients. In addition to CEA, serum exosomal piR-823 was discovered to be a reliable biomarker of CRC (Tong et al. 2023). Expression levels of piR-004800 in exosomes derived from bone marrow supernatant in MM patients and cells were associated with disease staging, which suggests that piR-004800 may be a marker for MM disease progression (Ma et al. 2020). Exosomal piR-25783 stimulates the TGF-β/SMAD2/3 pathway and encourages the secretion of different cytokines that play a significant role in the premetastatic microenvironment in metastatic ovarian carcinoma (Li et al. 2023). A total of 253 differentially expressed piRNAs were identified by small RNA sequencing analysis of piRNAs obtained from serum exosomes of HCC patients. These piRNAs were effective in differentiating HCC patients from non-tumor donors, suggesting that exosomal piRNAs could be potential diagnostic biomarkers for HCC (Rui et al. 2023). Exosomal piR-17560 produced by senescent neutrophils promotes the production of obesity-related protein in BC cells and provides tumor cells chemoresistance, suggesting that senescent neutrophils could be a viable target for therapy in the recurrence of BC (Ou et al. 2022). Exosomal piR-1089 is considered a quick, early, and easy noninvasive tool for neuroblastoma assessing metastasis. piR-1089’s mechanism in neuroblastoma cell proliferation and migration could provide more reliable experimental evidence for precision medicine (Wang et al. 2023). Exosomal piR-164586, piR-26925, and piR-5444 were significantly overexpressed in the serum of lung adenocarcinoma patients compared to healthy controls (Li et al. 2022). Recently, exosomes have been studied as potential nanocarrier therapies for a variety of diseases, including neurological conditions like Parkinson’s and Alzheimer’s. Exosomes’ small dimension allows them to pass through the BBB and into the CNS through a systemic mode of delivery. Exosomes allow Alzheimer’s disease cells to discard non-functional proteins, acting as “waste bags” in the process. Additionally, their RNA cargo can support the development of neural cells where piRNA and more precisely piR-823 would have potential worth testing.^2^ In this promising area, more studies are required to clarify the association between piRNAs and cancers.

### Therapeutic insights into piRNA

Several studies investigated the therapeutic applications of piRNAs in addition to their function as biomarkers. Despite the limited knowledge of the molecular pathways underlying piRNA activity in carcinogenesis, piRNA functional characteristics would make them excellent candidates for therapeutic purposes (Chen et al. 2021). To silence PIWI genes and eliminate negative consequences, specific piRNAs could be designed to bind PIWI proteins at the pre-transcriptional level. The PIWI/piRNA pathway may be a helpful tool for regulating the DNA methylation of particular genes at the transcriptional level, which leads to the transcriptional silencing of various oncogenes (Jia et al. 2022). DNA methylation is highly interesting for therapeutic approaches since it is reversible, unlike genetic modifications. By binding to mRNAs, piRNAs may prevent the generation of cancer-related proteins at the post-transcriptional level. That is similar to the process by which miRNAs can function without Dicer. Interestingly, studies have shown that piRNAs can bind to other proteins in addition to PIWI proteins. Being able to bind proteins offers a way for therapeutic intervention. Since piRNAs are both a novel target for future therapeutic drug discovery or repurposed medicine targeting^3^ and an efficient biomarker for cancer diagnosis, tumor gene therapy may advance due to ongoing research into specific interventions targeting piRNA expression. In both in vitro and in vivo studies, piRNA-targeted-therapy in conjunction with traditional chemotherapy has shown promising anticancer results. The combination of chemotherapeutic drugs such as paclitaxel, doxorubicin (DOX), and piR-54265 inhibitors in CRC or a piR-36712 analog in BC was more effective than treating with each agent alone (Mai et al. 2018; Tan et al. 2019).

#### Nano-bio-medicine (NBM) application

Recently, nanoparticle (NP) drug delivery systems (NDDSs) have become the most common system for co-delivering chemotherapy drugs and siRNA, which can inhibit the expression of genes associated with cancer or drug efflux transporters (Xiao et al. 2017). However, piRNA-based nano-formulations are still rarely used in the clinical field despite their promising potential application, and further studies are strongly required to develop new strategies to identify new barriers that hinder the use of RNA molecules in modern medicine. NPs can be classified into various classes according to their structure and features: polymeric, inorganic, lipid, and liposomal nanoparticles, and some of these types have proven to be convenient carriers for piRNAs (Han et al. 2024). The incorporation of specific piRNAs or piRNA inhibitors into the nanoparticle system has enabled their effective delivery to the targeted cells for the treatment of various cancer types. A nano-sized system of antago-miR-155 and piR-30074 with diethyl aminoethyl-dextran methyl methacrylate copolymer (DDMC) vector has achieved effective targeting and delivery of the bio-molecules to lung cancer cells, indicating a new therapeutic tool for lung cancer gene therapy (Klimenko 2017). Liposomal and polydopamine NPs of an encapsulated piR-1742 inhibitor suppressed the progression and metastasis of RCC. These NPs dramatically lowered the expression of MUC12 and piR-1742, implying the therapeutic potential of targeting piR-1742 in the treatment of RCC (Zhang et al. 2023). A magnetic NP-based gene therapy pre-loaded with anti-piR-823 in human BC cells transplanted in a mice xenograft model effectively suppressed carcinogenesis and tumor growth (Ding et al. 2021). The application of magnetic NP preloaded with piR-2158 in a BC xenograft model markedly inhibited the growth of mammary tumors, proliferation, and angiogenesis of breast cancer cells (Zhao et al. 2023). Gold nanoparticles (AuNPs) are inorganic nanoparticles most frequently used in various biomedical applications, including the detection and diagnostics of biomolecules. The AuNP system was developed as a fluorescent biosensing nano model to detect exosomal piR-823 ratiometrically in CRC samples (Sun et al. 2022). Taken together, treatment with a NP system pre-loaded with both chemotherapeutic drugs and piRNA mimic or inhibitor opens a new horizon in cancer therapy and is worth future exploration.

## piR-823 contributing to cancer-hallmark

**Fig. 6:**
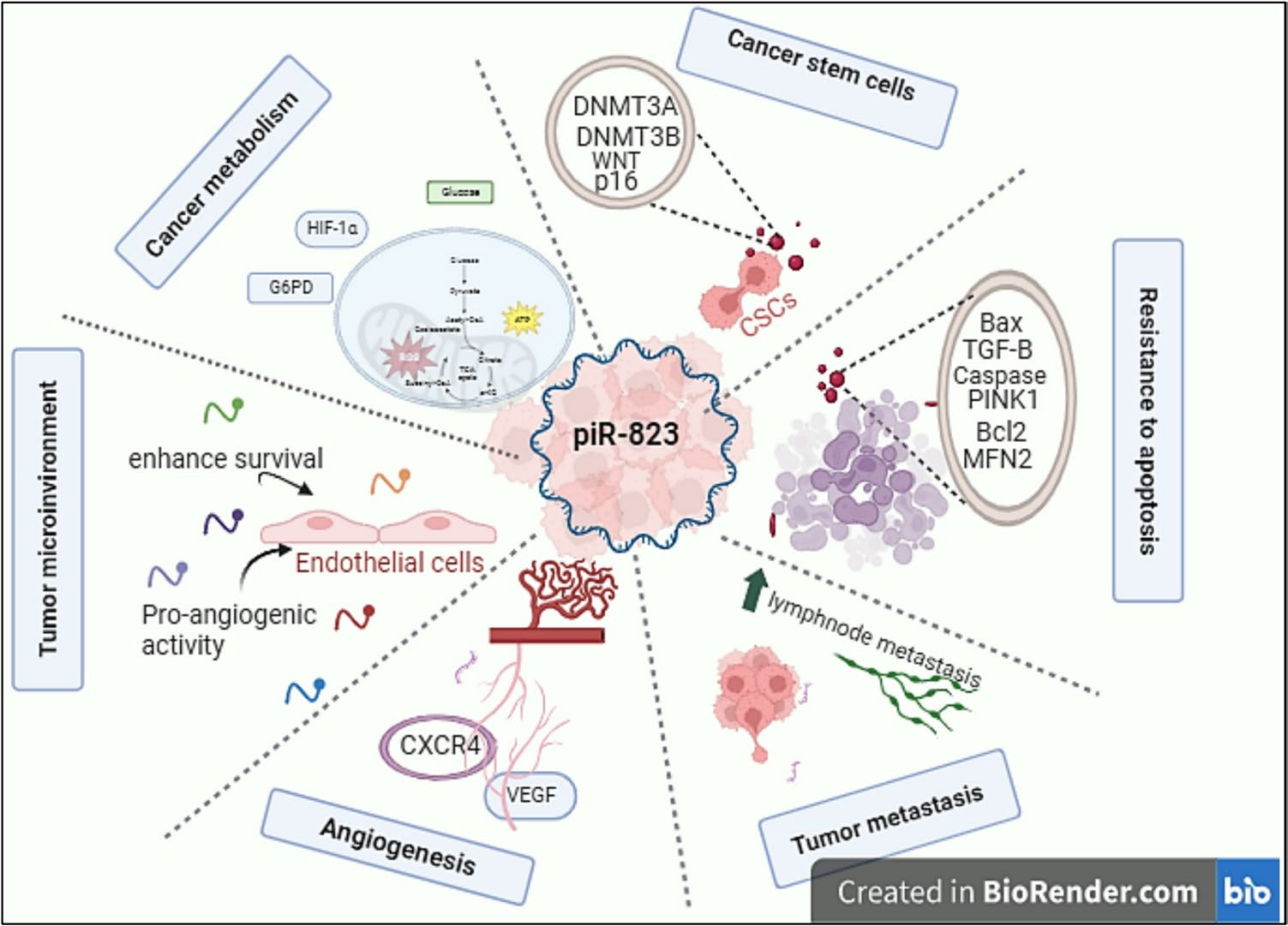
piR-823 contribution in various cancer hallmarks. piR-823 involved in cancer biogenesis through regulating multiple cancer hallmarks such as cancer stem cell, cancer metabolism, tumor microenvironment, angiogenesis, metastasis, and apoptosis resistance (figure was created with BioRender.com). DNMT, DNA methyl transferase; TGF-1β, transforming growth factor-1beta; VEGF, vascular endothelial growth factor; CXCR4, C-X-C motif chemokine receptor 4; Bax, Bcl-2-associated X protein; Bcl2, B-cell lymphoma; WNT, wingless-related integration site; PINK1, PTEN-induced kinase1; MFN2, mitofusin2; G6PD, glucose-6-phosphate dehydrogenase; HIF‐1, hypoxia inducible factor-1; CSC, cancer stem cell

Cancer is often classified as a complex disease with multiple hallmarks, including cancer stem cells, resistance to apoptosis, angiogenesis, metastasis, tumor micro environment, chemoresistance, and cancer metabolism. As piRNAs are deregulated in virtually all human cancers, they show involvement in each of the cancer hallmarks as well. In this part, we describe the involvement of piRNAs especially piR-823 in cancer from a cancer hallmarks (Fig. 6).

### Cancer stem cells

With their ability to self-renew and differentiate, CSCs are a small and distinctive source of cells that can produce a wide range of tumor-forming cell types. They may also be the primary cause of drug resistance, metastasis, recurrence, and tumor progression (Zhao et al. 2023). Besides germline cells, piRNAs may have an extended function in regulating CSCs; however, the precise functions of piRNAs remain mostly unknown (Huang et al. 2013b). Through the upregulation of piR-823 expression and the activation of DNMT3B, G-MDSCs have provided MM cells with stem-like characteristics. The elevated levels of CSC-related genes and proteins in MM cells caused by G-MDSCs are reversed by piR-823 silencing. Considering this, piR-823 appears to be an essential mediator in the maintenance of MM stem cells (Ai et al. 2019). Furthermore, piR-823 controlled DNA methylation to promote stemness and proliferation of breast CSCs, indicating that DNA methylation patterns are a mechanism for piRNA-mediated carcinogenesis and offer an in-depth comprehension of the various epigenetic patterns of stem cells and somatic cells (Ding et al. 2021).

### Resistance to apoptosis

Resistance to apoptosis is one of the major cancer hallmarks contributing to tumor occurrence, progression, and chemoresistance (Modabber et al. 2023; Mohammad et al. 2015). In recent years, PIWI/piRNAs have taken part in the process of apoptosis (Tan et al. 2022). piR-823 regulates apoptosis in a diversity of cells. In MM, the overexpression of piR-823 significantly attenuates apoptosis through inhibition of caspase-3 activation, downregulation of Bcl-2-associated X protein (Bax), and upregulation of Bcl2 (Yan et al. 2015). The two most significant mitophagy receptors, parkin and PINK1, are tumor suppressor genes involved in inducing apoptosis, establishing a significant association between mitophagy pathways and apoptosis (Bernardini et al. 2017). Accordingly, inhibiting piR-823 promoted apoptosis in CRC cell lines in a way dependent on mitophagy. In piR-823-silenced cells, cleaved PARP, cleaved caspase-9, and cleaved caspase-3 protein levels decreased due to parkin inhibition (Wang et al. 2022b). Inhibiting piR-823 in CRC tissues induces cell apoptosis, causes cell cycle arrest in the G1 phase, and reduces the progression of CRC (Yin et al. 2017). Various studies have shown that the TGF-1β signaling pathway plays a key role in the cell apoptosis. The combination of piR-823 and EIF3B-mediated TGF-1β expression in HSC promotes liver fibrosis and cirrhosis (Tang et al. 2018).

### Tumor metastasis and invasion

The primary feature of malignant tumors is metastasis, which is also a crucial indicator of tumor progression and a poor outcome. DNA methylation, histone alteration, and piRNA aberrant expression are among the epigenetic changes attributed to cancer metastasis. Comprehending the piRNA-related epigenetic pathways of tumor metastasis might help in the finding of novel tumor markers and therapeutic interventions (Liu et al. 2018a). By comparing benign, metastatic, and non-metastatic kidney tissue, 19 piRNAs were identified to be differentially expressed, and 46 were substantially related to metastasis. Three piRNAs (piR-32051, piR-39894, and piR-43607), among the metastasis-related piRNAs, were associated with metastatic renal carcinoma of advanced tumor stage and cancer-specific characteristics in patients (Li et al. 2015). piR-823 and PIWI protein expression levels have been correlated to TNM staging in renal cancer (Slaby et al. 2016). Furthermore, the TNM stage and distant metastases in GC were positively associated with the piR-823 level (Cui et al. 2011). Consequently, the prognosis of the disease and the TNM stage can be predicted using piRNAs as biomarkers. Moreover, piRNAs may act as a switch that permits tumor proliferation, spread, and metastasis.

### Tumor angiogenesis

Angiogenesis is a critical phase in the development and progression of tumors, which promotes invasive tumor proliferation and distant metastasis (Teleanu et al. 2019). Only a few studies explored the role of piRNAs, specifically piR-823, in tumor angiogenesis (Chen et al. 2021). In MM cells, the VEGF pathway may have a paracrine angiogenic role that promotes the development of tumors. piR-823 could promote carcinogenesis in MM by controlling the release of VEGF (Yan et al. 2015).

### Tumor microenvironment (TME)

Because the tumor microenvironment offers a rich environment for exploring novel therapeutic targets, it is essential to the invasion and metastasis of tumors (Elanany et al. 2023). Tumor cells may create such as microvesicles and exosomes to interact with other stromal cells by transferring ncRNAs that has the potential to alter the tumor microenvironment and promote tumor growth (Zhu et al. 2019). A favorable environment for MM may be created by piR-823 supplied by MM-derived EVs, which may improve the survival and angiogenic function of the ECs (Li et al. 2019).

### Cancer metabolism

Another apparent cancer hallmark is the way energy is metabolized in cancer cells. According to recent research, cancer cells can modify their glucose metabolism and shift toward aerobic glycolysis by increasing glycolysis in the presence of oxygen and inhibiting the tricarboxylic acid cycle (Pavlova and Thompson 2016). Thus, key features of human cancer that distinguish it apart from normal tissues include excessive energy production and metabolic reprogramming, which are controlled by several oncogenes and tumor suppressors (Dong et al. 2020). The suppression of HIF-1α ubiquitination by upregulating G6PD expression can be achieved by upregulating piR-823 in CRC cells. This can lead to an increase in glucose uptake and a decrease in intracellular ROS levels in cancer cells (Yin et al. 2017). A novel type of pro-inflammatory-programmed cell death known as hyperglycemic-induced pyroptosis can be inhibited by upregulating piRNA-823 expression in endothelial cells (Tan et al. 2024).

## Future prospective from the expert opinion perspective

Besides all the addressed research gaps, the current comprehensive information about piRNA, particularly piR-823, in various tumors opens the door for future prospective studies experimentally via knocking down piR-823 and its axis of related genes to prove the concept of utilizing piRNA directly to inhibit the transcription of a specific down-stream gene or multiple genes or proteins, mediated through other epigenetic alteration(s). More thorough research at the cellular and animal levels on the precise mechanism by which piRNA identifies its target in the genome and the possibility of off-target effects would be required to accomplish such an objective (Sun and Han 2019). Further studies are required to explore the relation of piR-823 with adipokines SNPs (Aboouf et al. 2015), or adipokines expression (El-Mesallamy et al. 2013), inflammation molecular markers, namely, the tumor immune microenvironment, insulin resistance or insulin-like growth factors (El-Mesallamy et al. 2012), and related hormones or genes in various cancer types. Moreover, future research to link piRNA SNP variants or haplotypes in different types of cancer will help identify clinical cancer cases at an early stage or low grade, ensuring personalized earlier diagnosis for better reflection on improving patients’ survival. Consequently, a step toward ncRNA precision is to implement ncRNA measurements in liquid biopsy or tumor tissues in addition to the epigenome project.

### Limitations

Compared to miRNAs, using piRNAs in the clinical field is still in its infancy, with limited information about piRNAs’ role in various non-communicable diseases, including different cancer types or rare diseases. Moreover, the possibility that many piRNAs could originate from the same genomic region, known as the piRNA cluster, adds another layer of difficulty to the mechanistic investigation of piRNAs; therefore, examining such clusters is a necessity.

## Conclusion(s)

It should not be too long until piRNAs are introduced into diagnostic, prognostic, and therapeutic clinical applications, given the amount of data that has been obtained regarding piRNAs for the sake of ncRNA use in precision medicine. piR-823 has become a promising cancer-specific biomarker due to its aberrant expression in several cancers. However, further research is still needed to fully understand the tumor-suppressive or carcinogenic functions of piR-823. Identifying the molecular mechanistic functions of piR-823 in various cancer hallmarks is likely to provide more insights into their roles in cancer initiation, progression, and metastasis. Interestingly, the presence of piRNAs in the exosomes suggests the potential application of piRNAs for distant target sites delivered along with drugs via exosomes or nano-biomedicine.

## Data Availability

No datasets were generated or analysed during the current study.

## Footnotes

1 research gap no. 1.

2 research gap no. 2.

3 research gap no. 3.

## Abbreviations

3′ UTR: 3′ Untranslated region
Ago: Argonaute3
Aub: Aubergine
BC: Breast cancer
Bcl: B-cell lymphoma
CDK: Cyclin-dependent kinase
CDS: Protein-coding sequence
CEA: Carcinoembryonic antigen
CSCs: Cancer stem cells
DNMT: DNA methyl transferase
DOX: Doxorubicin
ECs: Endothelial cells
EIF3B: Eukaryotic initiation factor 3 B
ER: Estrogen receptor
EVs: Extracellular vehicles
G-MDSCs: Granulocytic-MDSCs
G6PD: Glucose-6-phosphate dehydrogenase
GC: Gastric cancer
GI: Gastrointestinal
GWAS: Genome-wide association study
HCC: Hepatocellular carcinoma
Her2: Human epidermal growth factor receptor 2
HIF-1 α: Hypoxia-inducible factor-1-alpha
HSCs: Hepatic stellate cells
HSF1: Heat shock factor 1
LncRNA: Long non-coding RNA
MDSCs: Myeloid-derived suppressor cells
miRNA: MicroRNA
MM: Multiple myeloma
MMP: Matrix metallopeptidase
mRNA: Messenger RNA
mTOR: Mammalian target of rapamycin
ncRNA: Non-coding RNA
NP: Nanoparticle
nt: Nucleotide
PCR: Polymerase chain reaction
PINK: PTEN-induced kinase1
piRNAs: PIWI-interacting RNAs
PIWIL: PIWI-like protein
PR: Progesterone receptor
RCC: Renal cell carcinoma
RISC: RNA-induced silencing complex
ROS: Reactive oxygen species
siRNA: Small interfering RNA
SNP: Single-nucleotide polymorphism
STAT3: Signal transducer and activator of transcription 3
TEs: Transposable elements
TGF-β: Transforming growth factor beta
TGS: Transcriptional gene silencing
TNM: Tumor node metastasis
VEGF: Vascular endothelial growth factor
